# Impact of “Time-From-Biopsy-to-Prostatectomy” on Adverse Oncological Results in Patients With Intermediate and High-Risk Prostate Cancer

**DOI:** 10.3389/fsurg.2020.561853

**Published:** 2020-09-25

**Authors:** Tobias Engl, Philipp Mandel, Benedikt Hoeh, Felix Preisser, Mike Wenzel, Clara Humke, Maria Welte, Jens Köllermann, Peter Wild, Marina Deuker, Luis A. Kluth, Frederik C. Roos, Felix K. H. Chun, Andreas Becker

**Affiliations:** ^1^Department of Urology, University Hospital Frankfurt, Frankfurt am Main, Germany; ^2^Urogate Associates, Frankfurt am Main, Germany; ^3^Department of Pathology, University Hospital Frankfurt, Frankfurt am Main, Germany

**Keywords:** prognosis, waiting time, delayed treatment, deferred treatment, histological outcomes, radical prostatectomy, prostate cancer

## Abstract

**Objective:** Many patients with localized prostate cancer (PCa) do not immediately undergo radical prostatectomy (RP) after biopsy confirmation. The aim of this study was to investigate the influence of “time-from-biopsy-to- prostatectomy” on adverse pathological outcomes.

**Materials and Methods:** Between January 2014 and December 2019, 437 patients with intermediate- and high risk PCa who underwent RP were retrospectively identified within our prospective institutional database. For the aim of our study, we focused on patients with intermediate- (*n* = 285) and high-risk (*n* = 151) PCa using D'Amico risk stratification. Endpoints were adverse pathological outcomes and proportion of nerve-sparing procedures after RP stratified by “time-from-biopsy-to-prostatectomy”: ≤3 months vs. >3 and < 6 months. Medians and interquartile ranges (IQR) were reported for continuously coded variables. The chi-square test examined the statistical significance of the differences in proportions while the Kruskal-Wallis test was used to examine differences in medians. Multivariable (ordered) logistic regressions, analyzing the impact of time between diagnosis and prostatectomy, were separately run for all relevant outcome variables (ISUP specimen, margin status, pathological stage, pathological nodal status, LVI, perineural invasion, nerve-sparing).

**Results:** We observed no difference between patients undergoing RP ≤3 months vs. >3 and <6 months after diagnosis for the following oncological endpoints: pT-stage, ISUP grading, probability of a positive surgical margin, probability of lymph node invasion (LNI), lymphovascular invasion (LVI), and perineural invasion (pn) in patients with intermediate- and high-risk PCa. Likewise, the rates of nerve sparing procedures were 84.3 vs. 87.4% (*p* = 0.778) and 61.0% vs. 78.8% (*p* = 0.211), for intermediate- and high-risk PCa patients undergoing surgery after ≤3 months vs. >3 and <6 months, respectively. In multivariable adjusted analyses, a time to surgery >3 months did not significantly worsen any of the outcome variables in patients with intermediate- or high-risk PCa (all *p* > 0.05).

**Conclusion:** A “time-from-biopsy-to-prostatectomy” of >3 and <6 months is neither associated with adverse pathological outcomes nor poorer chances of nerve sparing RP in intermediate- and high-risk PCa patients.

## Introduction

There are numerous therapeutic options available for treatment of localized prostate cancer (PCa). Patients and their treating physicians can choose between active surveillance if the criteria are met, open or minimally invasive radical prostatectomy (RP), external radiotherapy, brachytherapy or, under certain circumstances, focal therapy (1). This decision can be very difficult for some patients. Many, especially critical and informed patients need time to make their decision and often ask for a second or third opinion. For this reason, some patients may have a significant delay in treatment, while other patients may be operated on promptly. Further potential reasons for delayed treatment include patient's anxiety, desire to obtain more detailed information regarding therapeutic options, necessity for treatment of comorbidities or limited surgical capacities and long waiting lists. Most studies show no influence of the time from diagnosis to surgery of low or intermediate risk prostate cancer on the oncological outcome (1–16). However, a recent Canadian multicentre study observed a higher risk of biochemical recurrence (BCR) in high-risk PCa patients undergoing RP after more than 3 months of waiting time (17). In the context of inverse stage migration with a trend toward surgical treatment of high-risk PCa and the need for prioritization strategies during the current COVID-19 pandemic, these concerns seem even more relevant (18).

To address this void, we investigated the impact of the time-from-biopsy-to-prostatectomy on histopathological outcomes and chance for nerve sparing surgery in men who underwent RP for intermediate- and high-risk PCa according to the D'Amico classification (19).

## Materials and Methods

### Study Population

Patients who underwent RP between January 2014 and December 2019 were retrospectively identified form our prospective institutional database (University Hospital Frankfurt, University of Frankfurt, Frankfurt am Main, Germany). All patients had given written consent and the study was approved by the local institutional review boards of the University Cancer Centre Frankfurt and the Ethical Committee at the University Hospital Frankfurt.

**Figure 1:**
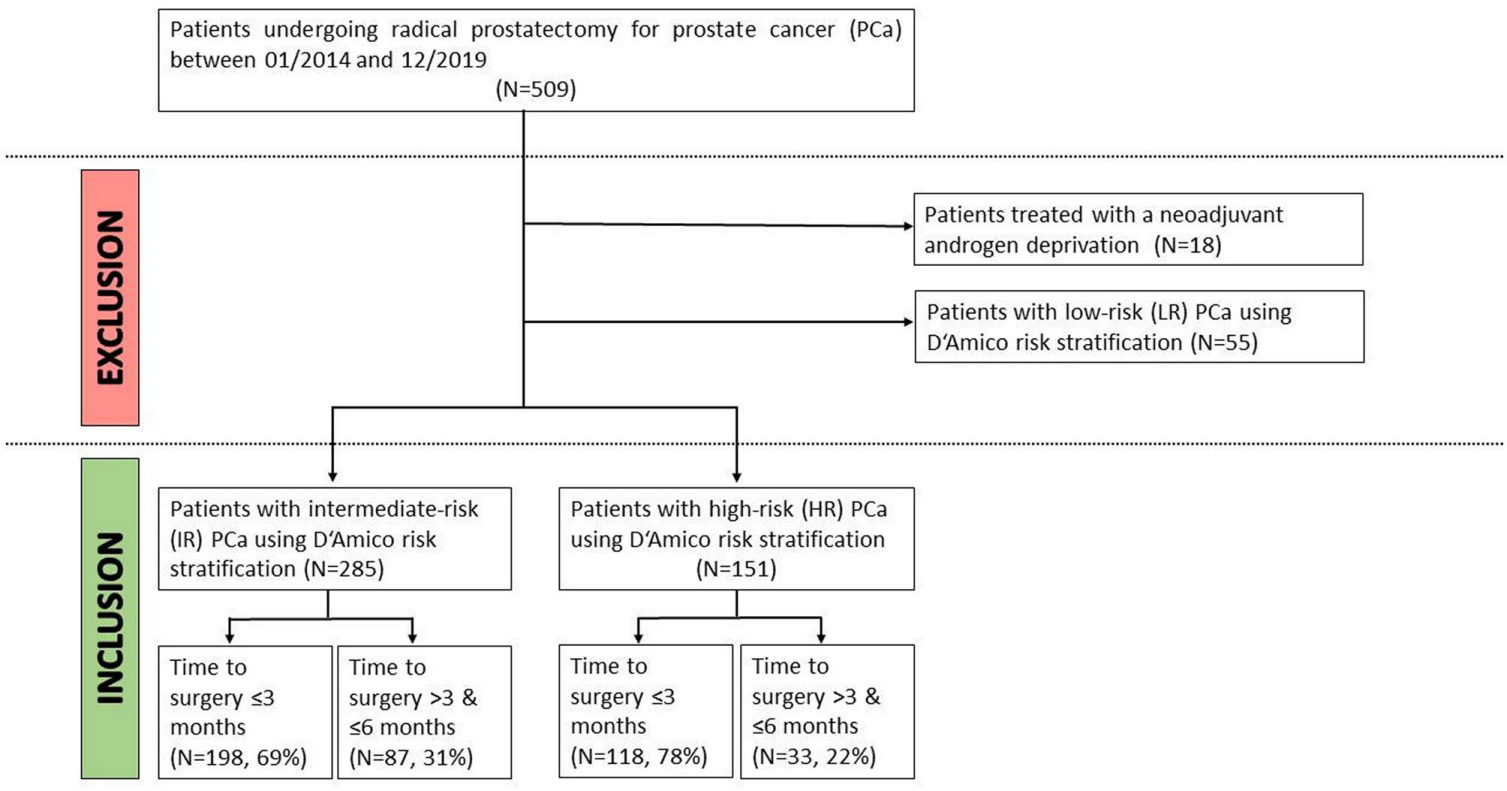
Inclusion- and exclusion criteria for our study of patients undergoing immediate (≤3 months) vs. delayed (>3 and <6 months) radical prostatectomy for intermediate- or high-risk prostate cancer.

Patients treated with a neoadjuvant androgen deprivation (*n* = 18, 3.5%) were excluded from our study, leaving 437 patients with intermediate- an high-risk PCa meeting the above-mentioned criteria (Figure 1). For the aim of our study, we focused on patients with intermediate- (*n* = 285) and high-risk (*n* = 151) prostate cancer using D'Amico risk stratification (19). Specifically, intermediate- vs. high risk PCa were defined as: PSA 10–20 ng/ml, Gleason sum >6, clinical stage ≥T2a, vs. PSA ≥20 ng/ml, Gleason sum 8–10, clinical stage >T2c).

Preoperative staging examinations were conducted according to the EAU guidelines: Lymph node imaging (using CT or MRI) and bone scans were conducted if PSA level >10 ng/mL or Gleason score >8 or clinical stage >T3 was present.

### Surgical Approach and Study Design

All patients underwent open retropubic or robotic-assisted laparoscopic RP. Each specimen was evaluated by two specialized uropathologists. Beginning in November 2017, radical prostatectomy was routinely performed with an intraoperative frozen section technique (NEUROSAFE) and with full functional length preservation of the prostatic urethra (FFLU) as described earlier (20, 21).

Patients were stratified by time from biopsy to surgery ≤3 months vs. >3 and <6 months. Relevant outcome variables were pathologic Gleason grade, pT-stage, surgical margin status, lymph node involvement (LNI), lymphovascular invasion (LVI), perineural invasion (PNI), and nerve sparing procedure. Stratified subanalyses were performed for patients with intermediate- and high-risk prostate cancer according to the D'Amico classification (19).

### Statistical Analyses

Descriptive statistics included frequencies and proportions for categorical variables. Medians and interquartile ranges (IQR) were reported for continuously coded variables. The chi-square test examined the statistical significance of the differences in proportions while the Kruskal-Wallis test was used to examine differences in medians. Moreover, multivariable (ordered) logistic regressions, analyzing the impact of time between diagnosis and prostatectomy, were separately run for all relevant outcome variables (ISUP specimen, margin status, pathological stage, pathological nodal status, LVI, perineural invasion, nerve-sparing). As control variables preoperative patient and tumor characteristics (age, BMI, prostate volume, PSA, cT stage, ISUP biopsy) were used. As statistical software STATA was used (version 14 for Windows, StataCorp LP, College Station, TX).

## Results

### Patient and Preoperative Tumor Characteristics

Patient and preoperative tumor characteristics stratified by time from diagnosis (biopsy) to radical prostatectomy are summarized in Table 1. Overall, 72.5% (*n* = 317) underwent surgery within 3 months from diagnosis while 27.5% (*n* = 120) underwent surgery between 3 and 6 months after diagnosis. There was no statistically significant difference according to age at surgery (*p* = 0.37), Body-Mass Index (BMI) (*p* = 0.57), prostate volume (*p* = 0.68), cT-Stage (*p* = 0.47), preoperative PSA (*p* = 0.54), and D'Amico classification (*p* = 0.06) between patients of the two subgroups. Only the biopsy ISUP Gleason grade grouping showed a significant difference between the two groups (*p* = 0.03).

**Table 1:** Preoperative characteristics of intermediate- and high-risk patients undergoing radical prostatectomy within 3 months after diagnosis compared to >3 and ≤6 months after diagnosis.

	**All patients (*n* = 437)**	**Time to surgery ≤3 months (*n* = 317)**	**Time to surgery >3 and ≤6 months (*n* = 120)**	***p*-value**
Age, median (IQR)	67 (71–61)	66 (71–61)	67 (72–61)	0.37
BMI (kg/m^2^), median (IQR)	26.2 (28.7–24.0)	26.2 (28.7–24.0)	25.4 (28.6–24.2)	0.57
Prostate volume in ml, median (IQR)	38 (50–30)	38 (50–30)	40 (58–30)	0.47
cT-stage, %				0.64
cT1	46.5	46.4	46.7	
cT2	49.9	50.5	48.3	
cT3	3.7	3.2	5.0	
PSA (ng/ml), median (IQR)	89.0 (13.6–6.2)	9.0 (13.8–6.2)	8.9 (12.8–6.2)	0.54
ISUP biopsy, %				0.03
1	8.5	6.9	12.5	
2	45.8	42.925.2	53.3	
3	22.9	15.1	16.7	
4	14.4	9.8	12.5	
5	8.5		5.0	
D'Amico, %				0.06
Intermediate-risk	65.4	62.7	72.5	
High-risk	34.6	37.4	27.6	

### RP Histopathologic Characteristics

Respectively, 65.4 and 34.0.6% of the analyzed patients showed preoperative intermediate-risk and high-risk characteristics according to the D'Amico classification. We observed no difference between patients undergoing radical prostatectomy ≤3 months vs. >3 and <6 months after diagnosis in terms of pT-stage, grading, probability of a positive surgical margin (PSM), probability of lymph node metastasis (LNI), lymphovascular invasion (LVI), or perineural invasion (PNI). This holds true for both patients with intermediate- and high-risk prostate cancer.

For intermediate-risk patients undergoing RP after ≤3 vs. >3 and <6 months, the rates of a uni- or bilateral nerve sparing was 84.3 vs. 87.4% (*p* = 0.778). For high-risk patients undergoing RP after ≤3 vs. >3 and <6 months, the rates of a uni- or bilateral nerve sparing were 61.0 vs. 78.8% (*p* = 0.211).

In multivariable adjusted analyses, a time to surgery >3 months did not significantly worsen any of the outcome variables in patients with intermediate- or high-risk PCa (all *p* > 0.05) (Table 2).

**Table 2a:** Histopathological outcomes and rates of nerve sparing surgery for patients with intermediate risk prostate cancer according to D'Amico.

	**All patients (*n* = 285)**	**Time to surgery ≤3 months (*n* = 198)**	**Time to surgery >3 and ≤6 months (*n* = 87)**	***p*-value**
ISUP specimen (%)				0.664
1	9.0	8.6	9.7	
2	58.5	60.1	55.3	
3	23.9	23.7	24.3	
4	3.7	2.5	5.8	
5	5.0	5.1	4.9	
Margins status (%)				0.242
R0	79.7	77.8	83.5	
R1	20.3	22.2	16.5	
Pathological stage (%)				0.996
≤pT2c	66.8	66.7	67.0	
pT3a	26.2	26.3	26.2	
≥pT3b	7.0	7.1	6.8	
Pathological nodal status (%)				0.650
pN0	96.0	96.0	96.1	
pN1	4.0	4.0	3.9	
Lymphovascular invasion (%)	7.6	8.1	6.8	0.211
Perineural invasion (%)	68.1	67.2	69.9	0.593
Nerve-sparing (%)				0.778
Unknown	1.4	1.5	1.1	
None	13.3	14.1	11.6	
Uni-/Bilateral	85.3	84.3	87.4	

**Table 2b:** Histopathological outcomes and rates of nerve sparing surgery for patients with high risk prostate cancer according to D'Amico.

	**All patients (*n* = 151)**	**Time to surgery ≤3 months (*n* = 118)**	**Time to surgery >3 and ≤6 months (*n* = 33)**	***p*-value**
ISUP specimen (%)				0.233
1	2.7	2.6	3.1	
2	30.2	28.2	37.5	
3	20.1	19.7	21.9	
4	16.8	20.5	3.1	
5	30.2	29.1	34.4	
Margins status (%)				0.164
R0	60.7	63.6	50.0	
R1	39.3	36.4	50.0	
Pathological stage (%)				0.144
≤pT2c	35.1	35.6	33.3	
pT3a	32.5	28.8	45.5	
≥pT3b	32.5	35.6	21.2	
Pathological nodal status (%)				0.066
pN0	74.0	70.3	87.5	
pN1	26.0	29.7	12.5	
Lymphovascular invasion (%)	32.7	33.1	31.3	0.851
Perineural invasion (%)	80.7	78.0	90.6	0.245
Nerve-sparing (%)				0.211
Unknown	4.6	5.9	0	
None	30.5	33.1	21.2	
Uni-/Bilateral	64.9	61.0	78.8	

## Discussion

Due to the current COVID-19 pandemic, prioritization, and delay of oncological surgeries represents a major contemporary issue, raising concerns regarding oncological safety of such delay not only in the academic community but also in many patients and their treating physicians (22).

In the context of intermediate- and high-risk prostate cancer, our present study analyzing 437 patients with D'Amico intermediate- and high-risk prostate cancer who underwent prostatectomy after <3 months or 3 and <6 months after biopsy, implies two important messages.

First, a treatment delay of 3–6 months does not impair histopathologic outcomes. We observed no difference in terms of pT-stage, grading, probability of a positive surgical margin, probability of lymph node involvement (LNI), lymphovascular invasion (LVI), or perineural invasion (PNI).

Second, we observed that using a frozen section guided approach (NEUROSAFE) (20) the probability to undergo nerve-sparing surgery is also not affected by such treatment delay. Over 87% of patients with intermediate-risk and almost 80% of patients with high-risk PCa received uni- or bilateral-nerve sparing delayed RP.

Our results are in contrast with a recent study from Zanaty et al. who observed a higher risk of biochemical recurrence (BCR) in high-risk PCa patients undergoing RP after more than 3 months of waiting time (17). However, mean waiting time in this Canadian series of 619 patients with low-, intermediate-, and high-risk prostate cancer was more than 5 months, whereas two thirds of patients in the present study underwent RP within 3 months after diagnosis. Moreover, in the Canadian study, no information on histopathologic characteristics such as extraprostatic extension (ECE), LNI was available. As such it seems possible that the observed correlation between waiting time and risk of BCR is due to very long waiting times and inclusion of a large proportion of patients with very high-risk patients harboring non-locally confined PCa.

However, most available studies investigating this topic reported no significant influence of delayed prostatectomy on oncological outcomes (5, 7, 11, 12, 17, 23). For example, Morini et al. recently published a study examining the impact in patients with localized prostate cancer reporting no correlation in time-from-biopsy-to-prostatectomy and impaired pathological results or risk of BCR (15). In another recent study by Gupta et al. these results could be confirmed. Specifically, there was no significant difference in rates of adjuvant therapy, PSM, ECE, seminal vesicle invasion, LNI or 2- and 5-year BCR-free survival between men who underwent radical prostatectomy at <3 months vs. 3–6 months after diagnosis (24). Also, Abern et al. observed that a waiting time of up to 9 months was not related to higher risk of BCR, ECE, PSM or histopathologic upgrading in their analyses of a large cohort of 1.561 men with low- and intermediate-risk prostate cancer undergoing radical prostatectomy (25). However, they observed an increased risk of BCR and PSM for patients with intermediate-risk prostate cancer who underwent radical prostatectomy at >9 months after biopsy. This observation confirms the current clinical practice to recommend definite therapy (i.e., radical prostatectomy or radiation therapy) for patients with intermediate-or high-risk prostate cancer (26).

Taken together we found no difference between prompt surgery and delayed surgery in neither the intermediate- nor the high-risk group in terms of histopathological characteristics or chance of nerve sparing in patients undergoing radical prostatectomy within 6 months.

Our results suggest that also in intermediate- and high-risk PCa patients oncological and functional outcomes after radical prostatectomy are unaffected by a delay of up to 6 months after diagnosis. Our findings might reassure patients with prostate cancer who cannot or do not want to be operated on immediately as well as their treating physicians. Our observations might be of special interest because of the ongoing discussion on treatment delay due to the current COVID-19 pandemic (27). Moreover, the focus on intermediate- and high-risk PCa patients accounts for the treatment paradigm shift in surgical PCa therapy, reflected by an inverse stage migration during the last decade allowing implications for current clinical practice (18).

Due to the retrospective nature of our study several limitations apply.

First, a selection bias leading to prioritization of patients with highest risk to immediate surgical treatment might deteriorate oncologic outcomes and probability of nerve sparing surgery. This circumstance could be an explanation for the fact that in the group that was operated on within 3 months the percentage of pT3b tumors and nodal positive patients was higher than in the group that that underwent delayed surgery.

Second, we were not able to determine the specific reason for the treatment delay. In this sense we do not know whether the patient was not operated due to anxiety, medical reasons, if the surgeon refused the prostatectomy initially or if the patient seeked a second opinion.

Third, our analyzed were restricted to clinico-pathologic characteristics. As such, we don't know about (biochemical) survival or functional results.

## Conclusion

Our data suggest that a delayed surgical treatment between 3–6 months does not affect pathological outcomes or the chance to obtain nerve sparing surgery in patients with intermediate- or high-risk prostate cancer.

## Data Availability Statement

The raw data supporting the conclusions of this article will be made available by the authors, without undue reservation.

## Ethics Statement

The studies involving human participants were reviewed and approved by University Cancer Center Frankfurt: SUG-4-2020. The patients/participants provided their written informed consent to participate in this study.

## Author Contributions

TE: concept of the study and writing the publication. PM: evaluation of the statistics, results, and correction of the publication. BH: material and methods, and illustrations. FP, MWen, CH, and MWel: database, and material and methods. JK and PW: evaluation pathology. MD: results. LK and FR: statistics and result. FC and AB: discussion and correction. All authors contributed to the article and approved the submitted version.

## Conflict of Interest

The authors declare that the research was conducted in the absence of any commercial or financial relationships that could be construed as a potential conflict of interest. The handling editor declared a past co-authorship with several of the authors LK, FC, and AB and the reviewer KB declared a past co-authorship with one of the author PM.

## References

[1] European Association of Urology Guidelines Prostate Cancer. (2020). Available online at: https://uroweb.org/guideline/prostate-cancer/ (accessed May 10, 2020).

[2] Sommers BD Beard CJ D'Amico AV Dahl D Kaplan I Richie JP. Decision analysis using individual patient preferences to determine optimal treatment for localized prostate cancer. Cancer. (2007) 110:2210–7. 10.1002/cncr.23028 17893907

[3] Song L Chen RC Bensen JT Knafl GJ Nielsen ME Farnan L. Who makes the decision regarding the treatment of clinically localized prostate cancer-the patient or physician? Results from a population-based study. Cancer. (2013) 119:421–8. 10.1002/cncr.27738 22786794 PMC7671233

[4] Holmes-Rovner M Montgomery JS Rovner DR Scherer LD Whitfield J Kahn VC. Informed decision making: assessment of the quality of physician communication about prostate cancer diagnosis and treatment. Med Decis Making. (2015) 35:999–1009. 10.1177/0272989X15597226 26304063

[5] Boorjian SA Bianco FJ Scardino PT Eastham JA. Urological oncology. Does the time from biopsy to surgery affect biochemical recurrence after radical prostatectomy? BJU Int. (2005) 96:773–6. 10.1111/j.1464-410X.2005.05763.x 16153197

[6] O'Brien D Loeb S Carvalhal GF McGuire BB Kan D Hofer MD. Delay of surgery in men with low risk prostate cancer. J Urol. (2011) 185:2143–7. 10.1016/j.juro.2011.02.009 21496847

[7] Graefen M Walz J Chun K-HF Schlomm T Haese A Huland H. Reasonable delay of surgical treatment in men with localized prostate cancer - impact on prognosis? Eur Urol. (2005) 47:756–60. 10.1016/j.eururo.2005.02.004 15925069

[8] Freedland SJ Kane CJ Amling CL Aronson WJ Presti JC Terris MK. Delay of radical prostatectomy and risk of biochemical progression in men with low risk prostate cancer. J Urol. (2006) 175:1298–303. 10.1016/S0022-5347(05)00646-4 16515984

[9] Phillips JJ Hall MC Lee WR Clark PE. Does a delay in initiating definitive therapy affect biochemical recurrence rates in men with clinically localized prostate cancer? Urol Oncol Semin Orig Invest. (2007) 25:196–200. 10.1016/j.urolonc.2006.06.004 17483015

[10] Nam RK Jewett MAS Krahn MD Robinette MA Tsihlias J Toi A. Delay in surgical therapy for clinically localized prostate cancer and biochemical recurrence after radical prostatectomy. Can J Urol. (2003) 10:1891–8. 12892576

[11] Kim SJ Ryu JH Yang SO Lee JK Jung TY Kim YB. Does the time interval from biopsy to radical prostatectomy affect the postoperative oncologic outcomes in Korean men? J Korean Med Sci. (2019) 34:e234. 10.3346/jkms.2019.34.e234 31559708 PMC6763398

[12] Khan MA Mangold LA Epstein JI Boitnott JK Walsh PC Partin AW. Impact of surgical delay on long-term cancer control for clinically localized prostate cancer. J Urol. (2004) 172:1835–9. 10.1097/01.ju.0000140277.08623.13 15540733

[13] Sun M Abdollah F Hansen J Trinh Q Bianchi M Tian Z. Is a treatment delay in radical prostatectomy safe in individuals with low-risk prostate cancer? J Sexual Med. (2012) 9:2961–9. 10.1111/j.1743-6109.2012.02806.x 22672479

[14] Westerman ME Sharma V Bailey GC Boorjian SA Frank I Gettman MT. Impact of time from biopsy to surgery on complications, functional and oncologic outcomes following radical prostatectomy. Int Braz J Urol. (2019) 45:468–77. 10.1590/s1677-5538.ibju.2018.0196 30676305 PMC6786103

[15] Morini MA Muller RL de Castro Junior PCB de Souza RJ Faria EF. Time between diagnosis and surgical treatment on pathological and clinical outcomes in prostate cancer: does it matter? World J Urol. (2018) 36:1225–31. 10.1007/s00345-018-2251-5 29549484

[16] Fossati N Rossi MS Cucchiara V Gandaglia G Dell'Oglio P Moschini M. Evaluating the effect of time from prostate cancer diagnosis to radical prostatectomy on cancer control: can surgery be postponed safely? Urol Oncol Semin Orig Invest. (2017) 35:150.e9–e15. 10.1016/j.urolonc.2016.11.010 27986374

[17] Zanaty M Alnazari M Ajib K Lawson K Azizi M Rajih E. Does surgical delay for radical prostatectomy affect biochemical recurrence? A retrospective analysis from a Canadian cohort. World J Urol. (2018) 36:1–6. 10.1007/s00345-017-2105-6 29052761

[18] Leyh-Bannurah S-R Karakiewicz PI Pompe RS Preisser F Zaffuto E Dell'Oglio P. Inverse stage migration patterns in North American patients undergoing local prostate cancer treatment: a contemporary population-based update in light of the (2012). USPSTF recommendations. World J Urol. (2019) 37:469–79. 10.1007/s00345-018-2396-2 29992380

[19] D'Amico AV Whittington R Malkowicz SB Fondurulia J Chen M-H Tomaszewski JE. The combination of preoperative prostate specific antigen and postoperative pathological findings to predict prostate specific antigen outcome in clinically localized prostate cancer. J Urol. (1998) 160:2096–101. 10.1097/00005392-199812010-00041 9817331

[20] Preisser F Theissen L Wild P Bartelt K Kluth L Köllermann J. Implementation of intraoperative frozen section during radical prostatectomy: short-term results from a German tertiary-care center. Eur Urol Focus. (2019) [Epub ahead of print]. 10.1016/j.euf.2019.03.007 30905598

[21] Schlomm T Heinzer H Steuber T Salomon G Engel O Michl U. Full functional-length urethral sphincter preservation during radical prostatectomy. Eur Urol. (2011) 60:320–9. 10.1016/j.eururo.2011.02.040 21458913

[22] Campi R Amparore D Capitanio U Checcucci E Salonia A Fiori C. Assessing the burden of nondeferrable major uro-oncologic surgery to guide prioritisation strategies during the COVID-19 pandemic: insights from three Italian high-volume referral centres. Eur Urol. (2020) 78:11–5. 10.1016/j.eururo.2020.03.054 32307215 PMC7151319

[23] Vickers AJ Bianco FJ Boorjian S Scardino PT Eastham JA. Does a delay between diagnosis and radical prostatectomy increase the risk of disease recurrence? Cancer. (2006) 106:576–80. 10.1002/cncr.21643 16353213 PMC1774862

[24] Gupta N Bivalacqua TJ Han M Gorin MA Challacombe BJ Partin AW. Evaluating the impact of length of time from diagnosis to surgery in patients with unfavourable intermediate-risk to very-high-risk clinically localised prostate cancer. BJU Int. (2019) 124:268–74. 10.1111/bju.14659 30570825

[25] Abern MR Aronson WJ Terris MK Kane CJ Presti JC Amling CL. Delayed radical prostatectomy for intermediate-risk prostate cancer is associated with biochemical recurrence: possible implications for active surveillance from the SEARCH database. Prostate. (2013) 73:409–17. 10.1002/pros.22582 22996686

[26] Mottet N Bellmunt J Bolla M Briers E Cumberbatch MG De Santis M. EAU-ESTRO-SIOG guidelines on prostate cancer. Part 1: screening, diagnosis, and local treatment with curative intent. Eur Urol. (2017) 71:618–29. 10.1016/j.eururo.2016.08.003 27568654

[27] Stensland KD Morgan TM Moinzadeh A Lee CT Briganti A Catto JWF. Considerations in the Triage of urologic surgeries during the COVID-19 pandemic. Eur Urol. (2020) 77:663–6. 10.1016/j.eururo.2020.03.027 32279903 PMC7146681

